# Supplementary material for: Correction: Trends in Inequalities in Health, Risk and Preventive Behaviour among the Advanced-Age Population in Austria: 1983-2007
Source: PLoS One. 2014 Aug 25;9(8):e107084. doi: 10.1371/journal.pone.0107084 (PMC4143351; doi:10.1371/journal.pone.0107084)
Supplement: File S1 — Originally published, uncorrected article. (PDF) [file pone.0107084.s001.pdf]

Trends in Inequalities in Health, Risk and Preventive Behaviour among the Advanced-Age Population in Austria: 1983-2007

Johanna Muckenhuber[1]*, Karina Fernandez[2], Nathalie T. Burkert[1], Franziska Groβschädl[1], Wolfgang Freidl[1], Éva Rásky[1]

1 Department of Social Medicine and Epidemiology, Medical University Graz, Graz, Austria, **2** Department of business education and development, Karl-Franzens-University Graz, Graz, Austria

Abstract

Background: Although a number of previous research studies have focused on the long-term analysis of the health and health behaviour of the elderly, there is still a shortage of information in relation to the long-term trends regarding health or risk and preventive behaviour in the elderly population taking into account gender differences and differences in educational level.

Methods: The database comprised subsamples of the Austrian Micro-Census, including individuals aged 65 years and older, for the years 1983, 1991, 1999, and subsamples of the ATHIS (Austrian Health Interview Survey) 2007. A trend analysis was conducted for four health-related variables with the year of the survey and education as predictors. The analysis was stratified by sex.

Results: We found a general trend towards better self-rated health, better preventive and less risk behaviour among the elderly, while the body mass index has been increasing over the years. There are indeed gender differences regarding the trend in smoking behaviour. While the prevalence of male smoking has been steadily decreasing, female smoking prevalence has not changed. At all points in time, individuals with higher education had significantly better self-rated health than those with lower education but the association between education and preventive behaviour significantly decreased over the years.

Conclusion: We agree with previous research in concluding that preventive action and health promotion should aim in particular to support older women and men with lower education.

Citation: Muckenhuber J, Fernandez K, Burkert NT, Groβschädl F, Freidl W, et al. (2014) Trends in Inequalities in Health, Risk and Preventive Behaviour among the Advanced-Age Population in Austria: 1983-2007. PLoS ONE 9(5): e97400. doi:10.1371/journal.pone.0097400

Editor: Gianluigi Forloni, "Mario Negri" Institute for Pharmacological Research, Italy

Received January 16, 2014; **Accepted** April 17, 2014; **Published** May 19, 2014

Copyright: © 2014 Muckenhuber et al. This is an open-access article distributed under the terms of the Creative Commons Attribution License, which permits unrestricted use, distribution, and reproduction in any medium, provided the original author and source are credited.

Funding: The authors have no support or funding to report.

Competing Interests: The authors have declared that no competing interests exist.

* E-mail: johanna.muckenhuber@medunigraz.at

Introduction

Compressing morbidity and maintaining health in the older population will be important challenges for public health activities during the next decades. Knowledge about trends in health, in preventive and in risk behaviour of older persons will be helpful in taking preventive action in order to improve health and decrease the prevalence of risk behaviour [1].

There is contradictory evidence regarding Fries' theory of compression of morbidity [2]. Empirical findings do not support recent compression of morbidity for the United States [3]. For the Austrian population Fries's theory has been confirmed and it has been argued that social changes and preventive efforts of the last decades have led to the compression of morbidity in late(r) life [4].

Research has found growing percentages of older people showing better preventive behaviour [5] and decreasing percentages for risk behaviour such as smoking [1]. By contrast, long-term trends of an increasing body mass index (BMI) have been observed [6,7]. It has been shown, that disability-free life expectancy has increased for more severe levels of disability or activity restrictions over the last decades [8].

Persons with low socio-economic status (SES) have poorer health than those with high SES [9]. This association is even stronger in the older than in the younger population [10].

Although a number of previous research studies have focused on the long-term analysis of the health and health behaviour of the elderly, there is still a shortage of information in relation to the long-term trends regarding health or risk and preventive behaviour in the elderly population taking into account gender differences and differences in educational level. In particular, there is a lack of knowledge concerning long-term trends in the association between educational level and self-rated health among the older population.

The aim of our study, therefore, was to investigate the long-term changes in self-rated health, in the prevalence of preventive and health behaviour, and in the strength of the association between

educational level and self-rated health, while also taking gender differences into account.

Data and Methods

The study was carried out in compliance with the declaration of Helsinki. The ethics committee of the Medical University of Graz approved this study

The database comprised two subsamples of the Austrian Micro-Census including i) individuals aged 75 years or older, and ii) individuals aged 65 years or older, for the years 1983 (N (65+) = 9217 (38.1% male)), 1991 (N (65+) = 8782 (36.7% male)), 1999 (N (65+) = 9416 (39.1% male)) plus two corresponding subsamples of the ATHIS (Austrian Health Interview Survey) 2007 (N (65+) = 3564 (41.5% male)). Face to face questionnaire interviews were conducted. Data was collected and provided by the Austrian Statistical Agency (Statistics Austria, 2011). The sample was representative for the Austrian population of the respective age-group.

Multivariate regression analyses were conducted concerning four health-related variables:

- Self-rated health (1 = very good, to 5 = very bad health)
- Vaccination against influenza (yes/no)
- Smoking habits (smoking yes/no) and
- Self-reported body mass index (bmi = kg/m^2).

We applied 4 models with linear regression analysis and 4 models with logistic regression analysis. In doing so, we used centred variables in order to avoid the common problem of multicollinearity, which can occur in regression models with interaction terms [11].

The year of the survey was integrated as dummy variable, with 1983 as category of reference.

In order to investigate the changing influence of "educational level" over time, we integrated the educational level and calculated interaction effects between the level of education and the year each survey was conducted. The level of education differentiated between compulsory education (nine years of schooling) and higher education. The analysis was stratified by sex.

Results

Our decision to analyse two subsamples came up during the study. At first we were mainly interested in persons of higher age (75 years or older). However, the subsample for this age group was rather small, so we decided to also include the subsample of 65 years and older, and to compare the results of the two age groups. We thus chose to describe the results for the group 75 years or older (see Table 1), and in cases of differing results to also describe results for the subsample of 65 years and older (see Table 2).

Trends in health and in male vs. female health behaviour

As shown in Figure 1 and in Tables 1 and 2, covering the years 1983 to 2007, we found a trend towards better self-rated health and improved health behaviour but also a higher BMI among older people for this period.

There was a continuous trend towards better self-rated health, with the best rating in 2007 for both older men (mean value (m): 2.72) and older women (m: 2.79) and the worst rating in 1983 (men, m: 2.99, women, m: 3.06). There was one exception, for men the difference in self-rated health between 1983 and 1999 is not significant.

The BMI increased significantly from 1983 (men, m: 24.35, women, m: 24.26) to 2007 (men, m: 25.55, women, m: 25.91). This increase was significant for men as well as for women, with

Table 1. Long-term-trends of health and health behaviour in persons 75 years and older (% yes, N, Mean Scores with SD, tests on significant differences between educational levels: Chi^2 and ANOVA) and trend in association (Spearman correlation rs and level of significance) between educational level and self-rated health.

	Vaccination	Smoking	BMI	health	Vaccination	Smoking	BMI	health
	Men: Logistic regression		Men: Linear regression		Women: logistic regression		Women: logistic regression	
	Exp(B)	Exp(B)	stand Beta	stand Beta	Exp(B)	Exp(B)	stand Beta	stand Beta
Level of Education	,523(,000/−1,80E+253)	1,517**(1,180/1,951)	−,039(−,575/,007)	−,145***(−,365/−,209)	,659(0,000/−1,80E+253)	,388***(,275/,546)	−,084***(−1,179/−,589)	−,147***(−,404/−,275)
Year 1983: category of reference								
Year_1991	,000(,000/−1,80E+253)	1,835***(1,362/2,472)	,051*(,038/,761)	−,080**(−,265/−,071)	,000(0,000/−1,80E+253)	,923(,594/1,434)	−,008(−,437/,288)	−,043*(−,165/−,007)
Year_1999	,254***(,194/,333)	2,201***(1,661/2,917)	,166***(,940/1,625)	−,012(−,117/,067)	,322***(,263/,392	,747(,500/1,115)	,107***(,638/1,284)	−,036*(−,142/−,001)
Year_2006	,161***(,121/,216)	3,524***(2,338/5,310)	,136***(,889/1,701)	−,065**(−,277/−,059)	,198***(,160/,245	1,254(,757/2,079)	,171***(1,561/2,288)	−,083***(−,287/−,127)
Year_1991*Edu	3,328(,000/−1,80E+253)	,889(,444/1,782)	,006(−,730/,929)	−,031(−,367/,079)	2,504(0,000//−1,80E+253)	,771(,284/2,094)	−,010(−1,078/,641)	−,019(−,274/,101)
Year_1999*Edu	1,535(,884/2,664)	1,260(,679/2,338)	,044(−,033/1,449)	−,014(−,259/,140)	1,799*(1,151/2,810	,494(,205/1,191)	,032(−,116/1,381)	−,008(−,201/,126)
Year_2006*Edu	2,571**(1,429/4,628)	,463(,207/1,038)	,059*(,218/1,901)	,000(−,226/,227)	2,036**(1,269/3,268	,748(,257/2,178)	,021(−,328/1,324)	−,002(−,190/,172)

Results of the four regression models, individuals 75 years old or older.
* p< = 0.05; ** p< = 0.005; *** p< = 0.001.
doi:10.1371/journal.pone.0097400.t001

Table 2. Long-term-trends of health and health behaviour in persons 65 years and older (% yes, N, Mean Scores with SD, tests on significant differences between educational levels: Chi^2 and ANOVA) and trend in association (Spearman correlation rs and level of significance) between educational level and self-rated health.

	Vaccination	Smoking	BMI	health	Vaccination	Smoking	BMI	health
	Men: Logistic regression		Men: Linear regression		Women: logistic regression		Women: logistic regression	
	Exp(B)	Exp(B)	stand Beta	stand Beta	Exp(B)	Exp(B)	stand Beta	stand Beta
Level of Education	,622(0,000/−1,80E+253)	1,444***(1,263/1,650)	−,008(−,238/,114)	−,167***(−,365/−,275)	,705(0,000/−1,80E+253)	,412***(,348/,488)	−,079***(−1,006/−,635)	−,160***(−,384/−,308)
Year 1983: category of reference								
Year_1991	,000(0,000/−1,80E+253)	1,533***(1,304/1,802)	,052***(,206/,644)	−,064***(−,188/−,077)	,000(0,000/−1,80E+253)	,871(,703/1,079)	,040**(,142/,594)	−,057***(−,156/−,063)
Year_1999	,308***(,261/,363)1,7977E)	1,671***(1,434/1,946)	,152***(,990/1,410)	−,007(−,068/,039)	,332***(,292/,378)	,865(,704/1,063)	,113***(,835/1,256)	−,037**(−,114/−,028)
Year_2006	,177***(,148/,213)	2,585***(2,079/3,214)	,150***(1,263/1,781)	−,043**(−,177/−,045)	,233***(,202/,268	1,197(,916/1,563)	,141***(1,496/1,991)	−,064***(−,218/−,115)
Year_1991*Edu	2,427(0,000/−1,80E+253)	,864(,594/1,257)	−,013(−,735/,260)	−,011(−,179/,074)	2,012(0,000/−1,80E+253)	1,012(,629/1,629)	,003(−,458/,603)	−,015(−,176/,043)
Year_1999*Edu	1,418*(1,011/1,989)	,808(,581/1,123)	,036*(,133/1,026)	,019(−,035/,192)	1,423*(1,066/1,900	,784(,504/1,219)	,019(−,097/,866)	,007(−,069/,130)
Year_2006*Edu	1,845**(1,280/2,660)	,604*(,391/,931)	,014(−,252/,798)	,008(−,094/,174)	1,641**(1,199/2,248)	1,155(,664/2,011)	,009(−,326/,773)	−,009(−,163/,064)

Results of the four regression models, individuals 65 years old or older.
* p< = 0.05; ** p< = 0.005; *** p< = 0.001.
doi:10.1371/journal.pone.0097400.t002

one exception. For the subsample of women 75 years or older, no significant increase in BMI between 1983 and 1991 was found; however, a significant increase in BMI appeared when comparing the year 2007 to the years 1991 and 1999.

The percentage of men and women vaccinated against influenza was significantly higher in 1999 (men: 30.6%, women: 26.9%) and in 2007 (men 38.8%, women 37.8%) when compared to 1983 (men: 7.2%, women: 8.3%). Such a significant increase was not found between 1983 and 1991, neither for men nor for women.

For men, we observed a significant decrease in the percentage of smokers, with the highest percentage in 1983 (23.3%) and the lowest percentage in 2007 (7.7%). For women, by contrast, we found neither increase nor decrease in smoking behaviour over the years (2.8% smokers in 1983, 2.7% in 2007).

Differences according to educational level

Table 1 shows that, consistently over the years, both men and women with high levels of education reported significantly better self-rated health than people with low levels of education.

Consistently over the years, women with low levels of education had a significantly higher BMI than women with higher levels of education. Leaving interaction effects aside, no such educational difference was found in men.

Setting interaction effects aside between the level of education and the year of the survey, significantly more older men with a low level of education were smokers compared to those with a high level of education. On the other hand, significantly more older women with high levels of education were smokers compared to those with a low level of education.

Changes over the years in the association between educational level and self-rated health

As shown in Figure 1, the relationship between the level of education and the percentage of men and women vaccinated against influenza changed significantly over the years. For both men and women, the educational difference played no significant role in 2007 when compared to 1983. For women, this also applies to the comparison between 1999 and 1983. In men, however, no such continuous pattern was found: the educational difference in respect of those being vaccinated against influenza was greater in 1999 than in 1983.

No significant interaction effects were found between the year of the survey and the educational effect on self-rated health and on the BMI. Consistently over the years, individuals with higher education had significantly better self-rated health than those with lower education, and also consistently over the years, women with a low level of education had a higher BMI than those with a high level of education.

Discussion

In accordance with previous research [2,4], our analyses have shown a trend towards better health among the older population. In line with other studies [9,12], our analyses have shown individuals with higher education to have better self-reported health than those with lower education. In accordance with some [12] though in contrast to other results [13], educational differences in self-rated health remained consistent over the years.

The persisting association between educational level and health among the older population might have two major causes. On the one hand, a low educational level is still associated with poor living standards [14]. Furthermore, with higher age, individuals have fewer possibilities to compensate for difficult living conditions such

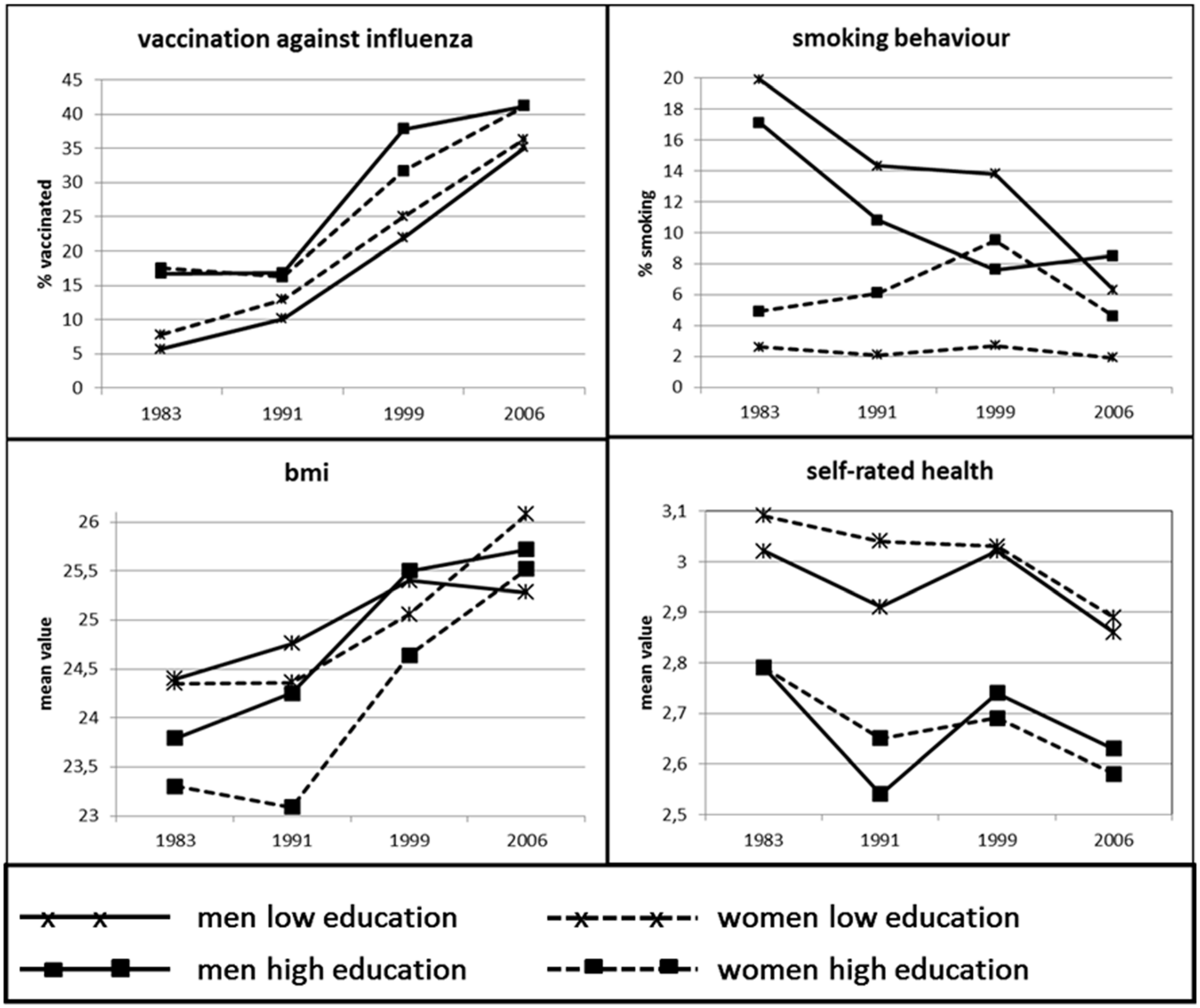

Figure 1. Long-term-trends of health and of health- and risk behaviour in persons 75 years and older.
doi:10.1371/journal.pone.0097400.g001

as insufficient heating etc. On the other hand, we have observed an increasing prevalence of preventive behaviour during the last decades. Even though we found the educational gap regarding preventive behaviour to have decreased over the years, preventive measures still reach population groups with higher education more easily and better than groups with lower education [15]. This might also have contributed to the persisting educational gap in health.

In line with some previous research [5,16,17] but in contrast to other research [18–20], we found increasing influenza vaccination coverage over the years. Regarding the case of Austria, we found very high rates of influenza vaccination within the older population as opposed to the general population. In contrast to previous research focussing on the general population [21], we found a steady increase in the proportion of older people vaccinated. In addition, we found decreasing educational differences over the years. These results might be explained by the fact that influenza vaccination is promoted by general practitioners in particular among the older population. Since older individuals with both higher and lower levels of education regularly see a G.P., this direct way of promoting the vaccination might have a particularly marked effect on the lower educated.

In accordance with some previous research on the general population [22,23] but in contrast to other studies focussing on older people [24], we found a trend toward increasing BMI among the older population.

In accordance with previous results, we found smoking prevalence in men to decline over the years [25,26] but no such decline can be reported for older women. This might be an effect of the age cohort with constantly low smoking prevalence (under 10%) in older women.

In contrast to previous research reporting lower percentages of smokers amongst both men and women with higher levels of education compared to those with lower levels of education [27–29], our study found a higher percentage of older women smoking within the higher educational level group compared to the lower educational level group. This might also be an effect of the age cohort. We assume that in the older female population smoking might be more readily accepted among the higher educated than among the lower educated women, since in the past smoking was

culturally less readily accepted for women than for men and even less accepted for women with a low level of education. We found different trends in smoking behaviour for men. In contrast to previous research showing greater declines in smoking among the higher educated than among the lower educated [29,30], our results show a greater decline in smoking prevalence for men with lower education than for men with higher education. This could be due to existing differences in the extent of smoking. Literature has shown heavy smoking to be more frequent among men with lower education than among men with higher education [31]. Possibly heavy smokers are more willing to stop smoking at a higher age than smokers who perceive themselves as light smokers.

Strengths and limitations

Strengths. Among the strengths of the study are the relatively high number of individuals included for analysis, the high data quality and the representative sample, the long time span analysed and the analysis of trends in differences regarding the educational level.

Limitations. One limitation of the study is that only a limited number of variables could be used for analysis and compared over the years because of the differing measures for health and for preventive and risk behaviour in the four data sets.

Another limitation is the measurement of "educational level". We had to divide education into basic low education and the wide category of high education, since there are very few women with higher education, in particular in the first years surveyed. When splitting the educational level into a greater number of categories, the number of older women with higher education turned out to be too small to perform a statistical analysis.

Conclusions. Our analysis has shown the enduring importance of the educational level for self-rated health over the years, so we agree with previous research [32] in concluding that preventive action and health promotion should in particular aim to support older women and men with lower education.

Author Contributions

Analyzed the data: JM KF NB FG WF ER. Wrote the paper: JM.

References

1. Marques-Vidal P, Cerveira J, Paccaud F, Cornuz J (2011) Smoking trends in Switzerland, 1992-2007: a time for optimism? J Epidemiol Community Health 65: 281–286.
2. Fries JF (1989) The compression of morbidity: Near or far? Milbank Memorial Foundation Quaterly/Health and Society 67: 208–232.
3. Crimmins EM, Beltran-Sanchez H (2011) Mortality and Morbidity Trends: Is There Compression of Morbidity? J Gerontol Ser B-Psychol Sci Soc Sci 66: 75–86. doi:10.1093/geronb/gbq088.
4. Doblhammer G, Kytir J (2001) Compression or expansion of morbidity? Trends in healthy-life expectancy in the elderly Austrian population between 1978 and 1998. Social Science and Medicine 52: 385–391.
5. Lu P, Singleton J, Rangel M, Wortley P, Bridges C (2005) Influenza vaccination trends among 65 years or older in the United States, 1989-202. Archives of internal Medicine 165: 1849–1856.
6. Grossschaedl F, Stronegger WJ (2013) Long-term trends in obesity among Austrian adults and its relation with the social gradient: 1973-2007. Eur J Public Health 23: 306–312. doi:10.1093/eurpub/cks033.
7. Staub K, Ruhli F, Woitek U, Pfister C (2010) BMI distribution/social stratification in Swiss conscripts from 1875 to present. European Journal of Clinical Nutrition 64: 335–340.
8. Cambois E, Clavel A, Romieu I, Robine J-M (2008) Trends in disability-free life expectancy at age 65 in France: consistent and diverging patterns according to the underlying disability measure. Eur J Ageing 5: 287–298. doi:10.1007/s10433-008-0097-1.
9. Freidl W, Stronegger W, Rasky E, Neuhold C (2001) Associations of income with self-reported ill-health and health resources in a rural community sample of Austria. Sozial- und Präventivmedizin 46: 106–114.
10. Chandola T, Ferrie J, Sacker A, Marmot M (2007) Social inequalities in self-reported health in early old age: follow-up of prospective cohort study. British Medical Journal 334: 990–993B.
11. Aiken L, West S (1991) Multiple Regression: Testing and interpreting interactions. Newbury Park, London, Delhi: Sage Publications.
12. Krokstad S, Kunst A, Westin S (2002) Trends in health inequalities by educational level in a Norwegian total population study. J Epidemiol Community Health 56: 375–380.
13. Dalstra J, Kunst A, Geurts J, Frenken F, Mackenbach J (2002) Trends in socioeconomic health inequalities in the Netherlands, 1981–1999. J Epidemiol Community Health 56: 927–934.
14. WHO Comission on Social Determinants of Health (CSDH) (2008) Closing the gap in a generation. Health equity through action on the social determinants of health. Final Report.
15. Stronegger W, Freidl W, Rasky E, Berghold A (1998) Educational status and resources for child care as predictors of TBE vaccination coverage in schoolchildren of an endemic area in Austria. Zentralblatt für Hygiene und Umweltmedizin 201: 437–445.
16. Kwong JC, Rosella LC, Johansen H (2007) Trends in influenza vaccination in Canada, 1996/1997 to 2005. Health reports/Statistics Canada, Canadian Centre for Health Information = Rapports sur la sante/Statistique Canada, Centre canadien d'information sur la sante 18.
17. Rodriguez de Azero M (2008) Trends in seasonal influenza vaccine distribution in the European Union: 2003-4 to 2007-8. Euro surveillance: bulletin Europeen sur les maladies transmissibles = European communicable disease bulletin 13.
18. Blank PR, Freiburghaus AU, Ruf BR, Schwenkglenks MM, Szucs TD (2008) Trends in Influenza Vaccination Coverage Rates in Germany over Six Seasons from 2001/02 to 2006/07. Med Klin 103: 761–768. doi:10.1007/s00063-008-1121-0.
19. Castilla J, Martinez-Baz I, Godoy P, Toledo D, Astray J, et al. (2013) Trends in influenza vaccine coverage among primary healthcare workers in Spain, 2008-2011. Prev Med 57: 206–211. doi:10.1016/j.ypmed.2013.05.021.
20. Jimenez-Trujillo I, Lopez-de Andres A, Hernandez-Barrera V, Carrasco-Garrido P, Santos-Sancho JM, et al. (2013) Influenza vaccination coverage rates among diabetes sufferers, predictors of adherence and time trends from 2003 to 2010 in Spain. Human vaccines & immunotherapeutics 9. doi:10.4161/hv.23926.
21. Kunze U, Bohm G, Groman E (2013) Influenza vaccination in Austria from 1982 to 2011: A country resistant to influenza prevention and control. Vaccine 31. doi:10.1016/j.vaccine.2013.08.050.
22. Loranta V, Tongleth R (2010) Obesity: trend in inequality. J Epidemiol Community Health 54: 637–638.
23. Zaninotto P, Head J, Stamatakis E, Wardle H, Midell J (2009) Trends in obesity among adults in England from 1993 to 2004 by age and social class and projections of prevalence to 2012. J Epidemiol Community Health 63: 140–146.
24. Kahn H, Cheng Y (2008) Longitudinal changes in BMI and in an index estimating excess lipids among white and black adults in the United States. International Journal of Obesity 32: 136–143.
25. Regidor E, De Mateo S, Ronda E, Sánchez-Payá J, Gutiérrez-Fisac J, et al. (2010) Heterogeneous trend in smoking prevalence by sex and age group following the implementation of a national smoke-free law. J Epidemiol Community Health online 2010.
26. Sardu C, Mereu A, Pitzalis G, Minerba L, Contu P (2006) Smoking trends in Italy from 1950 to 2000. J Epidemiol Community Health 60: 799–803.
27. Johnson W, Kyvik KO, Mortensen EL, Skytthe A, Batty GD, et al. (2011) Does Education Confer a Culture of Healthy Behavior? Smoking and Drinking Patterns in Danish Twins. Am J Epidemiol 173: 55–63. doi:10.1093/aje/kwq333.
28. Panasiuk L, Mierzecki A, Wdowiak L, Paprzycki P, Lukas W, et al. (2010) Prevalence of Cigarette Smoking among Adult Population in Eastern Poland. Annals of agricultural and environmental medicince 17: 133–138.
29. Smith P, Frank J, Mustard C (2009) Trends in educational inequalities in smoking and physical activity in Canada: 1974–2005. J Epidemiol Community Health 63: 317–323.
30. Giskes K, Kunst A, Benach J, Borrell C, Costa G, et al. (2005) Trends in smoking behaviour between 1985 and 2000 in nine European countries by education. J Epidemiol Community Health 59: 395–401.
31. Baumert J, Ladwig K-H, Ruf E, Meisinger C, Doering A, et al. (2010) Determinants of Heavy Cigarette Smoking: Are There Differences in Men and Women? Results From the Population-Based MONICA/KORA Augsburg Surveys. Nicotine Tob Res 12: 1220–1227. doi:10.1093/ntr/ntq172.
32. Stafford M, Nazroo J, Popay J, Whithehead M (2008) Tackling inequalities in health: evaluating the New Deal for Communities initiative. Journal of Epidemiology and Community Health 62: 298–304.
